# Parenting interventions for male young offenders: A review of the evidence on what works

**DOI:** 10.1016/j.adolescence.2011.10.007

**Published:** 2012-06

**Authors:** K. Buston, A. Parkes, H. Thomson, D. Wight, C. Fenton

**Affiliations:** MRC Social and Public Health Sciences Unit, 4 Lilybank Gardens, Glasgow G12 8RZ, UK

**Keywords:** Parenting interventions, Young offenders, Literature review, Evaluations, Fatherhood

## Abstract

Approximately one in four incarcerated male young offenders in the UK is an actual or expectant father. This paper reviews evidence on the effectiveness of parenting interventions for male young offenders. We conducted systematic searches across 20 databases and consulted experts. Twelve relevant evaluations were identified: 10 from the UK, of programmes for incarcerated young offenders, and two from the US, of programmes for young parolees. None used experimental methods or included a comparison group. They suggest that participants like the courses, find them useful, and the interventions may improve knowledge about, and attitudes to, parenting. Future interventions should incorporate elements of promising parenting interventions with young fathers in the community, for example, and/or with older incarcerated parents. Young offender fathers have specific developmental, rehabilitative, and contextual needs. Future evaluations should collect longer-term behavioural parent and child outcome data and should use comparison groups and, ideally, randomization.

## Introduction

There is a high rate of teenage fatherhood amongst incarcerated young offenders. At least one in four of the thirteen thousand (Berman, 2010; Northern Ireland Prison Service, 2011; Scottish Prison Service, 2010) incarcerated young offenders in the UK is an actual or expectant father (Macmillan, 2005; Mardon, 1996). Surprisingly, there are no comparative official statistics for young men in the general population in the UK, or indeed for non-incarcerated young offenders, but in the US only around one in twenty 16–21 year olds are fathers (Savio Beers & Hollo, 2009).

Men who become fathers at a young age tend to have an accumulation of risk factors: low social class, early risk behaviour including sexual activity and substance use, mental health problems, lack of social support, and low educational attainment (Barlow et al., 2011; Thornberry, Smith, & Howard, 1997). Furthermore, a number of problems are associated with the day-to-day realities of being a young father including financial hardship and instability of intimate relationships and the impact these may have on continued involvement with the child (Fagan, Bernd, & Whiteman, 2007; Lamay, Cashman, Elfenstein, & Felice, 2010; Quinton, Pollock, & Golding, 2002). Young fathers are unlikely to know much about child development or effective parenting skills (Barlow et al., 2011).

Problems associated with being a father at a young age are likely to be heightened by incarceration (Kazura, 2001; Nurse, 2000) Involvement, and even contact, with one’s child tends to become more difficult, and financial hardship can be exacerbated. Furthermore, offending young fathers are even more likely than their non-offending peers to have poor mental health, problems with literacy and numeracy, and to engage in risk behaviours (Golzari, Hunt, & Anoshiravani, 2006). They are more likely to have been in care, experienced violence or sexual abuse at home, and/or experienced problematic parenting themselves (Biggam & Power, 1998; Shannon & Abrams, 2007). They are likely to have experienced a lack of trust in personal relationships and with social support agencies, and to have received little support from these sources (Dudley, 2007; Tyrer, Chase, Warwick, & Aggleton, 2005). Young offender fathers are, in short, likely to be the most disadvantaged amongst young fathers and to face an even greater number of barriers to becoming engaged and involved fathers than their non-offending peers.

This suggests the need for parenting interventions for young offender fathers, to help them fulfil their roles as fathers, and improve outcomes for the child (Lundahl, Tollefson, Risser, & Lovejoy, 2008). Importantly, such programmes could also improve outcomes for the offender himself (Barlow et al., 2011).

Though parenting programmes have been widely implemented in British Young Offender Institutions for around twenty years, no review has been undertaken of their effectiveness, nor of the effectiveness of parenting programmes for non-incarcerated young offenders. This paper uses systematic literature searches and expert consultation to bring together and review studies which evaluate parenting programmes for male young offenders. We critically assess the findings of the evaluations identified, and conclude by discussing what effective parenting interventions might look like and how they should be evaluated in the future.

## Methods

### Literature searches

Preliminary searches were conducted to identify evaluations of parenting programmes for male young offenders. Terminology and definitions around ‘young offenders’ are country specific, and are used inconsistently even within countries. In Scotland, for example, Young Offender Institutions house 16–21 year olds and ‘young offender’ tends to refer to this age group, though the age of criminal responsibility is 12. In England, offenders between the age of 10 (the age of criminal responsibility) and 17 are usually referred to as ‘juvenile offenders’, with those aged 18-21 more consistently referred to as ‘young offenders’. In the United States, where the age of criminal responsibility varies between states, the youngest being 6 years, the term ‘juvenile offender’ appears to be generally used for non-adult offenders. For this review we included studies which use the term ‘young offender’ or ‘juvenile offender’ (whether or not they have ever been incarcerated), or which refer to men who are or have been incarcerated when aged between 16 and 21 years. The parenting programmes evaluated could be delivered in a Young Offender Institution (YOI), or similar, or in a community setting, including settings linked to the criminal justice system such as parole sites. It was required that findings focused specifically on young offenders.

A search strategy was developed in discussion with an information scientist (CF) who conducted the database searches. Table 1 shows the search terms used, in various combinations and in accordance with requirements for each database in terms of fields and filters used. Information on the construction of the search strategies is appended. There were no date limits to any of the searches. The geographical range included developed countries only. All search terms were specified in English. Table 2 lists the 20 electronic bibliographic databases that were searched.

All abstracts (*n* = 4100) retrieved by the searches were scanned by CF. Clearly irrelevant references (there were many relating to parents of young offenders) were discarded. KB read full texts of potentially relevant references (*n* = 59).

Once the first author (KB) was familiar with this literature she conducted searches on the World Wide Web using Google to identify unpublished grey literature or work in progress not already identified. Prison and parole related websites in the UK and US were explored, including those of support organisations and pressure groups in the criminal justice area and those known to be involved in prison-based interventions. Web-sites relating to fatherhood and to parenting were also examined (a full list of web-sites searched is available from the authors).

Lead authors of the included studies were contacted by e-mail, as were additional experts in the field either known to KB from her own research on young offenders (Buston & Wight, 2010; Buston, 2008, 2010) or identified through Google searches. This in turn led to further expert contacts. Altogether, 48 experts were contacted. They were asked whether:

• they were aware of any work in progress in the area
• they were aware of, or themselves had, any plans for further studies in this field
• there was any other expert they thought should be contacted.

In this correspondence KB outlined her impression of the state of the field, and asked each whether s/he agreed with this assessment. Correspondence was continued until no new names emerged and it became clear that no substantive work had been missed and no new directly relevant work was planned.

Finally, in order to complement the above strategies, more restricted, but systematic, literature searches were conducted to identify recently published evaluations of more general fathering programmes. This was so that the work on young offenders could be set in context, but also to ensure that no work specifically on young offenders existed amongst this more general literature. No more studies meeting our inclusion criteria were identified.

## Results

Thirteen papers, reports or book chapters were identified. These focused on 12 studies, dating from 1991, which involved evaluation of one or more parenting programmes for male young offenders (Boswell & Wedge, 2002; Caddle, 1991; Dennison & Lyon, 2001; Jarvis, Graham, Hamilton, & Tyler, 2004; Lindfield, 2009; Macmillan, 2005; Mardon, 1996; Meek, 2007; Nurse, 2002, 2005; Parra-Cardona, Wampler, & Sharp, 2006; Renton, 2002; Sherlock, 2004). The two references by Nurse referred to the same study. Twenty eight programmes were evaluated. The studies are represented in Table 3 with key characteristics relating to the site of the study/programme, nature of the programme evaluated, study design, participants in the evaluation, and findings outlined for each. Not all of the studies (nor publications) identified were primarily concerned with evaluation, but all reported some data evaluating a parenting programme for male young offenders. All but two of the interventions were with incarcerated offenders; the exceptions were interventions for paroled offenders. Ten studies were sited in the UK, two in the US.

### What has been evaluated?

#### Programme sites

Of the evaluations of YOI based interventions, four focused on a single intervention in a single YOI (Jarvis et al., 2004; Macmillan, 2005; Mardon, 1996; Meek, 2007); one focused on a single intervention delivered over a number of YOIs (Renton, 2002). The other YOI-based studies focused on between two and nine YOIs delivering different interventions (Boswell & Wedge, 2002; Caddle, 1991; Dennison & Lyon, 2001; Lindfield, 2009; Sherlock, 2004). One of the US studies evaluated a single intervention delivered at a single parole site (Parra-Cardona et al., 2006), the other a state-wide intervention delivered at numerous sites run by the youth justice body (Nurse, 2002, 2005). Most of the interventions consisted solely of classroom-based sessions, though Macmillan (2005) described a classroom-based course supplemented by clinics run by health visitors for the men and their visitors.

#### Programme development and delivery

The design and delivery of the programmes varied. The first authors of five of the publications were involved in designing and delivering the interventions described (Macmillan, 2005; Mardon, 1996; Meek, 2007; Parra-Cardona et al., 2006; Renton, 2002). Courses were usually led by prison tutors, with some sessions incorporating outside input from specialists such as sexual health outreach workers. Several authors commented that almost all those involved in delivering the course were female (Dennison & Lyon, 2001; Jarvis et al., 2004; Mardon, 1996). As a high proportion of the young fathers came from mother-only families, male involvement could have had the additional advantage of reinforcing messages about the importance of fatherhood (Dennison & Lyon, 2001).

Most of the courses appeared to have been developed in-house for the particular YOI in which they were delivered, though there were exceptions to this, including *Family Matters, Parentcraft,* and *Young Men as Fathers*. The *Family Matters* course, for example, operated by New Bridge Trust, was described as having been delivered in numerous UK YOIs since 1991 (Renton, 2002).

When information was available, courses comprised between four (Dennison & Lyon, 2001) and 16 sessions (Jarvis et al., 2004). Sessions tended to be two or three hours long. Most ran over a number of weeks with one session per week. However, Meek (2007) deliberately scheduled the course she described (and ran) to run intensively over full days for one week.

Those developing the courses usually assumed relatively low literacy levels, with written work non-existent or minimal. The letter writing component of Parra-Cardona et al.’s (2006) intervention is an exception. Class and/or group discussions were commonplace, with videos/DVDs sometimes shown, role plays and quizzes and other games played. Where information was available, classes were described as small, ranging from two participants (Lindfield, 2009) to 20 (Nurse, 2002, 2005)

#### Programme content

Extent of information about the interventions varied. Few of the studies contained an explicit statement about course objectives. Only Parra-Cardona et al. (2006) outlined the mechanisms by which the course objectives were expected to be met, referencing the Parent Management Training Model. Most courses appeared to be largely information based. They typically included information about pre-school child development, sexual health including contraception, pregnancy and birth, safety and first aid, child abuse, accessing support, legal aspects of being a father and financial responsibilities. Some courses had a small skills-based element, perhaps around basic childcare such as bathing and changing a baby, and play and disciplining. Most included class discussion, focussing on the men’s attitudes, particularly around issues such as the role of the father.

There were three exceptions to these standard, primarily information based courses: *Family Matters* included in Dennison and Lyon’s (2001), Lindfield’s (2009) and Renton’s (2002) evaluations; *Young Men as Fathers* (Nurse, 2002, 2005) which included a substantial number of skills based sessions, and the intervention described by Parra-Cardona et al. (2006) as having emotional, behavioural and cognitive goals.

Only two courses included substantive content relating to the men’s status as prisoners (Jarvis et al., 2004; Meek, 2007). For example: discussion on keeping in contact with children/(ex) partners whilst incarcerated and managing expectations regarding relationships with the child on release. Making storytelling video tapes to send to one’s child was incorporated into two courses (Jarvis et al., 2004; Sherlock, 2004). Macmillan (2005) described the only intervention that comprised a classroom parenting course supplemented by a clinic for the offenders and their visiting partner, parents and/or child(ren) focussing on ‘family issues’. Both interventions piloted and evaluated in Lindfield’s (2009) study tried to involve family members in sessions within the YOI. They did this by including visits by grandparents of the young offender’s child. One child and her mother were also involved in one of their programmes.

#### Course delivery

How curricula were delivered was investigated through interviews with providers (Dennison & Lyon, 2001; Jarvis et al., 2004; Lindfield, 2009; Nurse, 2002, 2005; Sherlock, 2004) or the inmates themselves (Caddle, 1991), and through observations (Jarvis et al., 2004).

All the UK programmes were voluntary. Some were open to all inmates, some prioritised actual and expectant fathers, and some were restricted to them. Slightly more interventions appeared to be open to all inmates rather than targeted towards fathers, though eligibility for participation is not always clear. For the two interventions sited in the US, men were selected and *required* to attend (Nurse, 2002, 2005; Parra-Cardona et al., 2006).Those attending the intervention described by Parra-Cardona et al. (2006) were paid $15 per session. In the UK those completing one of the courses described by Sherlock (2004) were rewarded with an extra, ‘bonding’, prisoner – child visit.

#### Evalution design

None of the programmes was evaluated through a randomised control trial (RCT), in which individuals or sites are randomly allocated to the intervention or to the controls, or using any design approaching it in methodological rigour. None had a comparison group. Only one study used a before and after design, collecting (quantitative) baseline data from the men before they received the course, and again afterwards, in order to gauge changes in knowledge and attitudes relating to fatherhood (Caddle, 1991). The remaining studies collected self-report data during and/or after delivery of the intervention, most either shortly after completion of the course or immediately following completion of particular sessions. Methods used were: in-depth interviews with participants (Boswell & Wedge, 2002; Dennison & Lyon, 2001; Lindfield, 2009; Parra-Cardona et al., 2006), evaluation sheets (Jarvis et al., 2004; Lindfield, 2009; Meek, 2007; Renton, 2002) or informal feedback to course tutors (Mardon, 1996). Dennison and Lyon’s (2001) work was unique in the UK in following up the men six months after their release and conducting in-depth interviews. In the US, Parra-Cardona et al. (2006) conducted in-depth interviews with their paroled sample at three time-points over a two month period. In some cases data were collected by those delivering the course (Macmillan, 2005; Mardon, 1996; Meek, 2007; Renton, 2002).

Sample sizes ranged from six young offenders (Lindfield, 2009; Parra-Cardona et al., 2006) to nearly 200 (Nurse, 2002, 2005), and the level of detail collected varied. Parra-Cardona et al.’s (2006) small study (*n* = 6) collected very detailed qualitative data about the young offenders’ responses to the parenting course, at different time-points. Nurse’s (2002, 2005) larger study (*n* = 200) had a broader approach but collected few quantitative data on young offenders’ experience of the parenting programme. Sample sizes in four studies were unclear or not reported.

Most of the studies used young offenders’ self-reported data, solely or primarily, to assess the effectiveness of the parenting course in terms of outcomes for them. Other measures of effectiveness included the proportion of visitors making use of a family oriented clinic (Macmillan, 2005), length of course waiting lists and drop-out rates (Mardon, 1996), and data from supplementary interviews with course leaders and five of the children’s mothers (Dennison & Lyon, 2001).

### What do the evaluations tell us?

The study designs are such that no impacts of the programmes have been identified. There are, however, other findings worth highlighting.

#### Acceptability

Several of the studies highlighted that the young offenders liked the parenting course they received and/or found it useful (Dennison & Lyon, 2001; Lindfield, 2009; Meek, 2007; Nurse, 2002, 2005; Parra-Cardona et al., 2006; Renton, 2002; Sherlock, 2004). None found the courses were unacceptable to the young offenders. Staff interviewed were also overwhelmingly positive about the parenting courses.

Interviews with young offenders and staff highlighted particularly successful components of the programmes. These included the making of storytelling DVDs, the provision of opportunities for discussion, and involvement of a range of prison and community workers (Lindfield, 2009; Parra-Cardona et al., 2006; Sherlock, 2004). The interviews also highlighted limitations. These included lack of information relevant to the men’s very young children; lack of acknowledgement of how imprisonment shaped the men’s parenting behaviour; not dealing with potential parenting difficulties on release (Jarvis et al., 2004; Sherlock, 2004); lack of involvement of other family members (Lindfield, 2009; Sherlock, 2004); brief length of the course; lack of publicity surrounding it; and lack of reward in terms of increased visits (Parra-Cardona et al., 2006; Sherlock, 2004).

#### Reported impact on parenting behaviour and view of parenting

Knowledge increases among participants were reported by several studies (Caddle, 1991; Jarvis et al., 2004; Nurse, 2005; Renton, 2002). Caddle’s (1991) pre and post intervention comparison, involving 37 men, found increased knowledge about child’s development, how fathers could help a child develop, and the effect a new baby was likely to have on personal lives and relationships. Caddle (1991) evaluated five, somewhat different, programmes and did not relate the results to individual programmes.

A number of studies identified changes in attitudes. Caddle (1991) found the most notable attitude change related to parental discipline, with a halving of those who equated discipline with ‘physical punishment’. There was also an increase in the number who believed discipline to be a matter of ‘non physical reinforcement’. Several other studies pointed to fathers changing their view on their role as parents. Following course attendance they said they felt more committed to their involvement with their child (Boswell & Wedge, 2002; Dennison & Lyon, 2001; Mardon, 1996; Parra-Cardona et al., 2006). Young offenders also reported learning parenting skills and techniques (Caddle, 1991; Nurse, 2002, 2005). However, most of the studies failed to provide any more objective measures on whether the course did, indeed, change the young offenders’ behaviour. The one exception to this were the five in-depth interviews carried out with mothers who reported that the course had made little or no difference to their partner’s involvement with the child (Dennison & Lyon, 2001).

#### Expert consultation

When a summary of the results reported above was sent to 48 experts, 26 replied. They included personnel working at the Scottish Prison Service Headquarters; staff working within prisons in the UK; directors of, and policy officers for, charities concerned with the welfare of young people and/or of prisoners; lead authors of the papers included in this review; academics working in the criminal justice field; and a Drug Control and Crime Prevention Officer at the United Nations Office on Drugs and Crime. They were based in the UK, US or Austria. None expressed surprise that only 12 relevant studies had been found. There was agreement that firm evidence on the effectiveness of parenting interventions for male young offenders was lacking. They agreed that there seemed to be tentative support for the finding that offenders seem to like parenting programmes, and, in the short term at least, knowledge could increase and attitudes could change following intervention. Experts also agreed that evaluative work undertaken was not particularly rigourous, with no work focussing on longer-term outcomes.

## Discussion

Above we show that parenting programmes for male young offenders have rarely been evaluated, and little is known about their effectiveness. Twelve studies were identified which evaluated parenting programmes for young male offenders. The type of evaluation designs used limits our ability to learn about impacts or changes in key outcomes. No programme has been evaluated using experimental methods, nor have comparison groups been used. Only one study compared outcomes pre and post intervention. Furthermore, evaluations have sometimes been led by those involved in designing and delivering interventions, raising the question of their objectivity. Most often outcomes have been assessed during or immediately after participation in the parenting programme. The evaluations have focused on acceptability and satisfaction with the programme. Longer term impacts on parenting and child outcomes have rarely been assessed.

However, programmers and policy makers should recognise that the absence of evidence of effectiveness is not evidence of ineffectiveness. The findings highlighted here are likely to be useful to those working in the field, even though they do not demonstrate the effectiveness of any single intervention. The studies suggest that: young offenders tend to like the courses, programme delivery is acceptable to staff involved, young offenders tend to report greater knowledge and positive attitudinal change around aspects of parenting following participation in such courses, and there are a number of constraints to the delivery of parenting interventions within prisons.

Given the absence of evidence of effectiveness of parenting interventions specifically with young offenders, what can be learned from parenting interventions more broadly? It is certainly well established that they can modify parenting behaviour (Barlow et al., 2011). Since young offender fathers share many developmental needs with their non-offending peers, it may be useful to borrow from effective parenting interventions for young fathers in the community. It might also be that effective parenting interventions for incarcerated adult parents could point to components that might work with younger imprisoned fathers.

Unfortunately, however, studies evaluating such interventions share similar methodological limitations to those reviewed here (Barlow et al., 2011; Loper & Tuerk, 2006). To find firmer evidence of effectiveness we have to cast the net wider to review interventions for fathers generally (Bronte-Tinkew et al., 2008; Moran, Ghate, & van der Merwe, 2004).

Reviews suggest that promising interventions for fathers are likely to: use theoretical approaches that have been effective in influencing parenting behaviours in other contexts; have concrete objectives; make use of skills based methods and provide opportunities for practice; use teaching methods and materials that are appropriate specifically for fathers; use individual and group work; personalise information given; be of sufficient length to cover core activities adequately (at least eight weeks); and, ideally, involve the other parent and children (Bronte-Tinkew et al., 2008; Moran et al., 2004). The reviews also suggest that the most promising delivery of such programmes will involve: teachers who believe in the programme and have experience of the client group; training for these teachers; a high staff-participant ratio; fidelity to the curricula; incentives for participants; and attention to keeping them engaged (Bronte-Tinkew et al., 2008; Moran et al., 2004). All of these promising elements could be reproduced in parenting interventions for young offenders, though working in parallel with the other parent and child(ren) is likely to be particularly challenging with incarcerated offenders (Lindfield, 2009). Crucially, though, young offenders’ particular developmental and contextual needs must be considered.

There are several broad issues to consider in making more tailored recommendations as to where to go next with parenting interventions for young offenders. First, it is notable that many of the prison based interventions evaluated here were open to *all* inmates. They did not specifically target fathers (and expectant fathers). The needs of those who are already fathers are, however, likely to be very different to the needs of those who may become fathers at some undetermined time in the future. Delivering an intervention relevant to both sets of men is likely to be difficult. Our first recommendation is that targeted interventions should be the way to proceed, and priority should be given to those who are already fathers. Tailoring to the specific client group is an important feature of successful interventions (Lipsey, 1999; Loper & Tuerk, 2006; Moran et al., 2004).

Second, the context of the delivery site should be acknowledged to a greater extent than has been the case in most existing interventions. For most of the interventions evaluated here the delivery site is the prison. Loper and Tuerk (2006) highlight that parenting from prison is substantially different from parenting on the outside so interventions should be built around this. As well as aiming to change offenders’ parenting practices once they have left the YOI, interventions should teach skills around contact, communication and constructive engagement, with the child and his/her principal caregiver, from the prison. For example, what can the young offender talk about in brief telephone conversations with his young child? Expectations should be managed around the nature of parent–child relationships when contact is not daily. For example, how can the young offender deal with disappointment when his toddler refuses to speak to him on a monthly visit? Post-release issues also need to be addressed. Advice around finding employment, as well as possible issues around housing and support, are likely to be salient (Loper & Tuerk, 2006). Linkages to aftercare services in the community are likely to be crucial (Shannon & Abrams, 2007).

Third, the ‘doubly disadvantaged’ status of those fathers who have offended, and who are young, should be acknowledged to a greater extent. While most programmes evaluated in the studies reviewed here recognise the likely low literacy levels amongst participants, the special needs of the men beyond this do not seem to have been acknowledged, on paper at least. Risk behaviours which may work against constructive parent - child engagement, such as violence and substance abuse, should be addressed and work should be done to raise levels of self-respect and self-esteem. Clearly one must be realistic about the possible scope of any one intervention, but holistic models need to be utilised (Fagan et al., 2007; Lamay et al., 2010; Loper & Tuerk, 2006; Quinton et al., 2002).

The fourth recommendation also concerns the broadening of aims around what parenting interventions for young offenders should do. There should be clearer objectives around improving the offenders’ lives so that they have more of a stake in the father–child relationship and have a ‘good life’ generally in order that they are less likely to reoffend (Andrews, Bonta, & Wormith, 2011; Murray & Farrington, 2008). *The Good Lives Model*, for example, is one of the most cited theoretical models of rehabilitation. It posits the attaining of friendship, enjoyable work, loving relationships, creative pursuits, sexual satisfaction, positive self-regard and an intellectually challenging environment as the primary goals for offender rehabilitation (Andrews et al., 2011). Parenting programmes might be seen as helping create such good lives for the young offenders. Lipsey and Wilson (1998) found that institutionalized offenders who received ‘human service interventions’ showed around a 36 per cent decrease in re-offending compared to controls. Interpersonal skills programmes involving training in social skills, aggression replacement, anger control and cognitive restructuring evidenced the best outcomes. Cost, time and scope constraints are pertinent but parenting interventions for young offenders should aim to do more than just improve the men’s parenting knowledge, attitudes and skills.

Fifth, interventions should provide, in an overarching way, alternative models of masculinity for the young offenders. Ideas of normative masculinity centring around risk taking, and possibly violent, behaviours, need to be replaced by models that equate hegemonic masculinity with being a good father. Research suggests that many young men, and young offenders, do desire to be good fathers (Buston, 2010; Reeves, 2006; Tuffin, Rouch, & Frewin, 2010). ‘Being a provider’ is a central component of this, again highlighting the need for interventions to give men skills and resources to find employment, but so is spending time with one’s child, taking him/her places, and loving him/her (Buston, 2010; Ross, Church, Hill, Seaman, & Roberts, 2010). Young offenders could be enabled to feel masculine by being involved and engaged fathers in these ways.

These five recommendations all highlight that future programmes should have further reaching aims and curricula than the interventions evaluated here. In the UK, the interventions highlighted here have all been delivered within the prison and have been voluntary. In the US, both interventions were delivered by parole services and were mandatory. Evidence as to ‘what works best’ is inconclusive, but perhaps programmers on both sides of the Atlantic should consider approaches not traditionally used. Research suggests that incarceration provides the young offenders with time to think, a liminal time during which they are particularly open to turning their lives around and altering their behaviours and identity (Buston, 2010; Shannon & Abrams, 2007). Prison-based interventions seem appropriate to capitalise on this. However, using the time immediately following the men’s release, whilst they are on parole, may still capitalise on this but may allow more involvement of the child and partner, and more flexibility generally in what can be covered and how (Loper & Tuerk, 2006). The young offenders may be more likely to attend a parenting intervention when they are, literally, a captive audience within the prison - the difficulties of engaging with young fathers in the community have been well documented (Moran & Ghate, 2005) – but making attendance at a parole based intervention mandatory would ensure participation.

There needs to be evidence that the next generation of interventions work, and understanding as to how and why they do so. The quality of evidence could be greatly improved by collecting longer term behavioural parent and child outcome data. This would allow assessment of changes in child maltreatment, father’s self-esteem, recidivism, and father–child relationships. The use of a control group would improve the ability to attribute reported impacts to the intervention by comparing changes in similar outcomes among those not participating in the intervention. The use of experimental designs such as RCTs would further improve the strength of evidence by controlling for background confounders at an individual and/or an institutional level.

Future intervention development should take account of the particular developmental, contextual and rehabilitative needs of young offenders. Programmes should move beyond aiming to improve parenting knowledge, attitudes and skills. The next generation of evaluative work must be able to identify programme impacts. Challenges abound in developing and evaluating new programmes, but this is a very important and underdeveloped area of enquiry in the criminal justice system.

## Funding

This work was supported by core funding by the UK Medical Research Council as part of the Sexual Health and Families Programme (WBS U. 1300.00.005).

## Figures and Tables

**Table 1:** Search terms used.

Adolescence	Juvenile offender
Adolescent	Juvenile offenders
Adolescent fathers	Juvenile probation
Adolescent parents	Male
Adolescents	Offend
Child	Offender
Children	Offenders
Children of incarcerated offenders	Parent
Children of prisoners	Parent training
Correctional institutions	Parenthood
Crime	Parenting
Detention	Parenting interventions
Education	Parents
Family intervention	Paternity
Father	Pregnancy
Father child relation	Prison
Fathers	Prisoner
Imprisonment	Prisoners
Incarceration	Prisoners/education
Intervention-programmes	Prisons
Juvenile delinquency	Teenage fathers
Juvenile delinquent	Young offender
Juvenile delinquents	Young offenders
Juvenile inmates	Young people
Juvenile justice	Youth

Construction of search statements • Three concepts were identified for searching purposes: fatherhood, offending; and training • Search statements were constructed by combining terms concerning one concept such as ‘fatherhood’, using the Boolean operator ‘OR’. • groups of terms were then combined using the Boolean operator ‘AND’. Example of a search statement “Father” OR “Fathers” OR “Father child relation*” OR “Parent*” OR “Parenthood” AND “Juvenile delinquency” OR “Juvenile delinquent*” OR “young offender*” AND “Education”OR “Family intervention” OR “Intervention* OR “Parent training”.

**Table 2:** Electronic databases searched.

ASSIA
Campbell collaboration
Cochrane reviews
Cinhal
EBM reviews
Embase
ERIC
Francis
IBSS
National Criminal Justice Reference Service Abstracts
National Criminal Justice Reference Service
Psychinfo
PubMed
SCIE
Social Policy and Practice
Socindex
Sociological Abstracts
The Kings College Evidence Network
Vera Institute of Justice database
WoK

**Table 3:** Key characteristics of the programmes evaluated: site, nature, study design, participants in the evaluation and findings.

Authors	Country of study/programme, programme(s) evaluated	Study design & key data sources	Participants in evaluation	Findings
Boswell and Wedge (2002)	3 courses in 3 UK YOIs. ‘Needs led’, largely info based. ‘Major eligibility criterion’ is fatherhood or expectant fatherhood. One course is 6 sessions, no other specific info.	Retrospective uncontrolled study. Interviews with young offenders following completion of course.	30 young offenders.	Most reported that it had changed the way they perceived their fathering role and expected this to impact on their children.
Caddle (1991)	5 courses, in 5 UK YOIs. All largely info based. 3 open to all inmates, 1 to expectant/actual fathers,1 to those ‘who have responsibility for young children’. Between 6 and 12 sessions delivered.	Pre and post course interviews re knowledge and attitudes, including behavioural vignettes, and about the teaching techniques used in relation to their needs. Information gathered on selection of inmates for course.	37 young offenders (20 of whom had children).	Men felt they had learned new parenting skills. Following course: improvement in knowledge of stages of a child’s development and ways in which this may be facilitated, increased understanding of effect baby may have on personal lives and relationships, attitudes to role of father in maintaining parental discipline changed. The courses attracted participants who might most immediately benefit from training since 2/3 of sample were already actual/expectant fathers. Course with large component of discussion, rather than written work, likely to be most effective.
Dennison and Lyon (2001)	9 ‘best established most comprehensive’ courses in 9 UK YOIs plus 4 ‘special interest’. Includes *Family Matters.* All largely info based. 4 or more sessions delivered. Minority of courses open only to fathers or expectant fathers, most open to anyone interested. Majority of courses delivered by part-time tutors employed by education contractor within prison, ‘a number’ brought in specialists to deliver particular sessions. One course delivered by community health pros and one community volunteers. Predominantly female teaching input.	Retrospective uncontrolled study. Interviews with expectant/actual fathers who had participated in course; follow-up interviews with sub-sample 6 months after release. Interviews with mothers of children and with course leaders.	62 young offender fathers/expectant fathers, 25 followed up 6 months post-release. 5 mothers of offenders’ child. Course leaders interviewed for 12/13 courses.	Men liked courses and said they had learned from them, especially the factual based elements. At follow up they considered they had retained a significant amount of the course content and were finding it helpful in their post-release parenting role. Mothers were less positive feeling course had made little/no difference to father’s involvement with child. Course tutors strongly motivated, thought course worthwhile but identified barriers.
Jarvis et al. (2004)	1 course (*Parentcraft*) delivered in 1 UK YOI. Open to all inmates. Includes storybook tape and other content specific to men as prisoners.16 weekly 3 h sessions. Led by lecturer employed by local college with outside input for some sessions, primarily female input.	Retrospective uncontrolled study. Observation of sub-sample of sessions, focussing on response of students to modes of delivery of course; end of session and end of course evaluation sheets for students; course documentation scrutinised, interviews with teaching staff focussing on approaches to teaching and learning.	Observation of sessions approx. six-weekly over a period of 18 months. No numbers provided for student evaluations or interviews with teaching staff.	Young offenders report increase in knowledge and understanding in area of parenting. Observations and staff point to importance of relating information to men’s needs in relation to age of child and context of imprisonment. Opportunities for discussion, respect for the men, caring and supportive attitude using personal narratives and practical activities are important.
Lindfield (2009)	2 courses piloted in 2 UK YOIs. Includes *Family Matters*. Both courses included visits involving family members. Open to actual and expectant fathers. *Family Matters* delivered by New Bridge, other course delivered by prison staff including members of healthcare team and colleagues based in community. One course delivered to 4 men, other to 2 men.	Retrospective uncontrolled study. End of session questionnaires and in-depth interviews with young offender parents and staff	6 young offender fathers/fathers to be, involved staff (no number given).	Men: positive about courses, enjoyed them, thought they should be routinely offered, in theory supported involvement of family members although was not always possible. Staff: involving community-base colleagues contributes positively to courses; support of senior managers and involvement of uniformed and wing staff is crucial to delivering courses successfully; should link with YOT parenting co-ordinators to support learning and support from the parenting; is desirable but very difficult to involve family members in courses.
Macmillan (2005) (Author developed and delivered intervention)	1 intervention in 1 UK YOI. Parenting course and family orientated weekly clinic for young offender parents, partners, children and grandparents where skills could be developed and support for fathers as prisoners and mothers living as single parents could be given. Family Day on completion of course for men to practice skills with child and other carer. Programme led by health visitors, aided by prison tutor.	Retrospective uncontrolled study. Number of visitors making contact at clinic.	N/A	70 per cent of visitors actively sought clinic staff out.
Mardon (1996) (Author developed and delivered course)	1 course in 1 UK YOI. Largely information based. 11 weekly sessions. Open to actual and expectant fathers. Tutor led with input from health visitor, social services and marriage guidance with input from male prison staff encouraged. 8 participants per course.	Retrospective uncontrolled study. ‘Informal feedback’ from participants to tutor relating to how programme has affected interaction with partner. Length of waiting lists and drop-out rates.	Based on unspecified amount of author observation and reported feedback from participants over 9 years.	Positive feedback from young offenders about developments in their relationships with children at visiting times. Long waiting lists and low drop-out rates.
Meek (2007) (Author developed and delivered course)	1 course in 1 UK YOI. Largely info based, includes content specific to men as prisoners. Open to all inmates, priority given to actual/expectant fathers. 5–11 participants. 10 sessions delivered over a week. Delivered by author with assistance from prison staff, outside input for specific sessions.	Retrospective uncontrolled study. Anonymous written course evaluation completed by participants at end of course.	75 young offenders.	All participants rated course as *very* or *fairly* useful.
Nurse (2002,2005)	1 course – *Young Men as Fathers* - delivered in facilities under the jurisdiction of the Californian Youth Authority, US. Largely skills based. Participation required of selected inmates, with fathers given priority. 12 weekly sessions. 15–20 participants. Delivered by community educators.	Retrospective uncontrolled study. Observation of parole parenting classes. Survey of paroled fathers in Northern California including question about helpfulness of classes, in-depth interviews with sub-sample where some raised issue of classes attended though was not asked about. Evaluative data on course is by product of study whose focus is on effect of incarceration and parole on young men’s relationships with their children.	Approx 40 parenting class sessions observed in 4 parole offices. Around 200 fathers who had participated in course surveyed, in-depth interviews with 20 (though not all will, necessarily, have completed parenting course).	Classes generally viewed positively and felt by men to be helpful. Men reported benefiting through learning background knowledge, mastering specific techniques and learning new behaviour patterns. Observations suggest young men benefit because they are forced to think about fatherhood and to learn some alternatives to their old behaviour patterns with children.
Parra-Cadona et al. (2006) (Lead author developed and delivered course).	1 course in 1 parole setting in US. Emotional, behavioural and cognitive goals. 6 sessions of 2 h. Participation required of selected parolees, all fathers. Maximum 4 participants. Led by first author (marriage and family therapy graduate student) and parent educator.	Retrospective uncontrolled study. Repeat interviews (*n* = 3) over 2 month period with teen fathers who had completed course, focussing on their experiences as group participants and ways group processes influenced their experiences as fathers.	6 participants	Men reported: liking and finding course useful, wanting it to continue beyond 6 sessions, an initial defensiveness against participating in the group which soon dissipated, interactions with group leaders and fellow participants facilitated disclosure of personal experiences and challenged their views of their role as fathers, every topic addressed was beneficial.
Renton (2002) (Author involved in developing and delivering course)	1 course – *Family Matters* – delivered in unspecified number of UK YOIs (course delivered 22 times). Includes skills based sessions. 6 sessions of 2 h. Group size 8–12. Run by New Bridge, includes session from sexual health outreach workers.	Retrospective uncontrolled study. Evaluation forms completed by young offenders. Author’s own views deriving from her involvement in running courses.	Course delivered to 209 prisoners but article does not specify how many filled in evaluation forms.	Men positive about course and report greater awareness of the practical and emotional issues surrounding fatherhood and far greater understanding of contraception, STDs and testicular cancer.
Sherlock (2004)	2 courses in 2 UK YOIs. Both largely information based. Course A includes video story initiative where fathers filmed reading stories from books, film sent to child. Bonding visit for inmates completing course. 6 full days over a week.Course B offered to all inmates.	Retrospective uncontrolled study. Focus groups with young offender parents, their friends, family and staff. At YOI A, 4 stages of data collection with staff and prisoners and 1 with families and at YOI B 4 stages of data collection with staff and prisoners and 3 with families (respondents not necessarily same) over 18 month period, covering range of family-related issues.	No numbers provided.	Course A: prisoner feedback that more publicity needed about parenting course, they only do course to receive a bonding visit, course very helpful; staff feedback that would be good to extend parenting course to whole YOI but staffing levels mean this is not possible, module needs to be included on parenting from a distance to acknowledge status of men as prisoners, not enough room for ‘reward’ bonding visits to be done every week, course needs to focus more on children under three, the storytelling element works well. Course B: prisoner feedback that partners should be able to see what is covered on parenting and if parenting course is completed they should be entitled to more visits; staff feedback that beneficial if partners had more information about course or could attend some of sessions.
